# A microencapsulated blend of botanicals supports weaning piglets during a lipopolysaccharide challenge by modulating liver inflammation and intestinal integrity

**DOI:** 10.1093/jas/skae277

**Published:** 2024-09-18

**Authors:** Andrea Bonetti, Benedetta Tugnoli, Federico Ghiselli, Grace Markley, Elizabeth Cooper, Andrea Piva, Chad H Stahl, Ester Grilli

**Affiliations:** Dipartimento di Scienze Mediche Veterinarie (DIMEVET), Università di Bologna, Bologna, Italy; Vetagro S.p.A., Reggio Emilia, Italy; Vetagro S.p.A., Reggio Emilia, Italy; Vetagro S.p.A., Reggio Emilia, Italy; Department of Animal and Avian Sciences, University of Maryland, College Park 20742, MD, USA; Department of Animal and Avian Sciences, University of Maryland, College Park 20742, MD, USA; Dipartimento di Scienze Mediche Veterinarie (DIMEVET), Università di Bologna, Bologna, Italy; Vetagro S.p.A., Reggio Emilia, Italy; Department of Animal and Avian Sciences, University of Maryland, College Park 20742, MD, USA; Vetagro Inc., Chicago 60603, IL, USA; Dipartimento di Scienze Mediche Veterinarie (DIMEVET), Università di Bologna, Bologna, Italy; Vetagro Inc., Chicago 60603, IL, USA

**Keywords:** botanicals, inflammation, liver, LPS challenge, pigs, weaning

## Abstract

This study examined the action of a blend of botanicals (**BOT**) against lipopolysaccharide (**LPS**)-induced inflammation on cultured hepatocytes and weaning piglets. In vitro studies examined HepG2 cells treated with BOT and challenged with *Escherichiacoli* LPS for 8 d. BOT treatment reduced IL-6 concentration in cell culture media across time (*P* < 0.05) and decreased pro-inflammatory cytokine expression on days 1 and 8 of experiment (TNFα, IL-1β; *P* < 0.05). BOT also increased the expression of antioxidant enzymes (GPX-2, SOD, CAT) on day 8 (*P* < 0.05), which was supported by lowered reactive oxygen species concentration after LPS challenge (*P* < 0.1). The in vivo study was conducted with 72 weaning pigs, allotted into 24 pens and divided into 3 groups: a negative control (**CTR−**, basal diet), a challenged control (**CTR+**) that received an intraperitoneal injection of *E. coli* O55:B5 LPS on days 14 and 16, and a challenged treated group which received a diet containing 1.5 g/kg of microencapsulated BOT (**BOT+**) for the whole duration of the study. Growth performance was determined weekly and, on days 21 (1 animal per pen) and 28 (remaining animals), pigs were sacrificed to collect liver and jejunal tissues. After the challenge, BOT+ pigs had increased BW on days 21 (*P* < 0.05) and 28 (*P* < 0.1) compared to CTR+. Similar improvements in average daily gain and FCR on days 14 to 21 (*P* < 0.05) and 21 to 28 (*P* < 0.1) were also seen in BOT+ group. In the liver, compared to CTR+ pigs, BOT+ pigs had downregulated expression of TLR-4, IL-6, IFN-γ on day 21 (*P* < 0.05), and TLR-4, TNF-α, IL-8 on day 28 (*P* < 0.05). BOT+ also increased GPX-2 expression on days 21 and 28 (*P* < 0.05), while also upregulating SOD-1 and SOD-2 on day 21 (*P* < 0.05) and CAT on day 28 (*P* < 0.05) compared to CTR+. In the jejunum, BOT+ reduced inflammation by affecting cytokine expression (*P* < 0.05) and increasing the expression of tight-junction proteins, ZO-1 on day 21 and CLD-1 on day 28 (*P* < 0.05). Furthermore, BOT+ pigs had lower crypt depth on days 21 (*P* < 0.1) and 28 (*P* < 0.05), and increased villi-to-crypt ratio on days 21 and 28 (*P* < 0.05). By day 28, BOT+ intestinal measurements were restored to values similar to the CTR−. Finally, BOT+ also reduced mast cell activation on day 21 (*P* < 0.05) compared to CTR+. Considering all the findings, BOT controlled inflammatory activation and oxidative stress in liver cells, enhanced intestinal integrity, and as a result improved the growth performance of weaning piglets challenged with LPS.

## Teaser

Stress at weaning leads to inflammation and oxidative damage, resulting in reduced growth performance and potential long-lasting health consequences. Many botanicals have been recognized to have beneficial functions relating to inflammatory and oxidative stressors. In this study, a blend of botanicals helped hepatocytes manage inflammation and oxidative stress, and had positive effects on intestinal morphology and integrity, thus ensuring improved growth parameters during an inflammatory challenge.

## Introduction

The liver is the animals’ metabolic center, governing countless reactions, coordinating complex chemical pathways, detoxifying undesired compounds, and playing a vital role in the response to stress (Trefts et al., 2017). Weaning represents a considerable source of stress to young pigs (Zheng et al., 2021). During the adaptation to the stressors, weaning piglets experience inflammation and oxidative stress as part of their physiological response, which in turn leads to a decline in growth performance (Campbell et al., 2013; Pohl et al., 2017; Nordgreen et al., 2020). The response of the intestinal tract to the stressors of weaning includes the impairment of tight-junctions activity, caused by inflammation, and the reduced ability of enterocytes to absorb nutrients (Lallès et al., 2007; Tang et al., 2022): these factors produce gastrointestinal and immune system maturation delays that have long-lasting implications for pig performance (Smith et al., 2010; Moeser et al., 2017).

Systemic inflammation and oxidative stress predispose piglets to other pathologies. This is frequently seen with pathogenic intestinal bacteria (Luppi, 2017). This is often the case of enterotoxigenic *Escherichia coli*, which causes diarrhea after releasing toxins that further damage piglets’ intestinal epithelium (Dubreuil et al., 2016). Impairment of gut barrier integrity, regardless of being caused by systemic inflammation or bacterial toxins, paves the way for a dramatic increase in the translocation of unwanted antigens and compounds, such as bacterial lipopolysaccharide (**LPS**) (Ghosh et al., 2020). Once LPS is translocated, it is immediately bound by LPS binding protein (**LBP**) (Park and Lee, 2013), and the complex is transported to the liver through the portal vein system. Once arriving at the liver, hepatocytes and resident macrophages recognize the LPS–LBP complex via their membrane CD14–TLR-4–MD2 receptor system (Park and Lee, 2013), and work jointly to detoxify LPS, while also triggering an inflammatory response that involves the release of cytokines, and the synthesis of reactive oxygen species (**ROS**), with the aim to further bolster the immune system defense against bacteria (Su, 2002; Robinson et al., 2016). Prolonged LPS translocation in the intestine has been shown to lead to increased concentrations of insulin in the bloodstream, which enhances immune cell glucose uptake to fuel inflammatory activation (Kvidera et al., 2017). Prolonged hyperinsulinemia may also lead to peripheral insulin insensitivity, the inhibition of lipolysis, and increased hepatic glycogenolysis and gluconeogenesis (Kvidera et al., 2017). Under these health-challenged conditions, these anabolic processes would be accomplished at the expense of skeletal muscle, which are catabolized to provide gluconeogenic substrates. This would have prolonged effects on animal performance and could lead to liver injuries and other metabolic diseases (Kick et al., 2011; Ghosh et al., 2020; Duan et al., 2023).

Botanicals are complex mixtures of bioactive compounds extracted from plants that have a long history of use in animal nutrition because of the sustainability of beneficial health effects (Rossi et al., 2020). Among these compounds, antimicrobial, antioxidant, anti-inflammatory, and immunomodulatory activities have been identified (Rossi et al., 2020; Abdelli et al., 2021). We have demonstrated the efficacy of several of these botanicals when employed alone (Toschi et al., 2020b, 2022; Bonetti et al., 2023a), or in selected combinations to improve gut health, inflammation, barrier function, and susceptibility to pathogens in vitro (Bonetti et al., 2023b). Therefore, we hypothesized that botanicals could have beneficial effects in other in vitro cell lines and in weaning pigs when they are challenged by an inflammatory status.

The goal of this study was to determine if a combination of botanicals (**BOT**) could control inflammation, oxidation, and overall stress in cultured hepatocytes and in weaning piglets. To address this question, we conducted in vitro and in vivo challenge models and assessed the benefits of BOT.

## Materials and Methods

All experimental procedures were reviewed and approved by the University of Maryland at College Park (UMCP) Institutional Animal Care and Use Committee (IACUC Reference: #R-JUL-22-33). Animal housing, care, and procedures were conducted in accordance with PHS Policy on Humane Care and Use of Laboratory Animals, Animal Welfare Act & Regulations (7 USC 2131 et. Seq.), USDA Animal Care Policy Manual, and the local policies set forth by UMCP IACUC.

### In vitro *experiments*

Unless otherwise specified, chemicals and cell culture reagents were provided by Merck KGaA (Darmstadt, Germany). The blend of botanicals (**BOT**) tested in the present study was a proprietary mixture corresponding to the natural extracts contained inside AviPower^®^5 (Vetagro S.p.A., Reggio Emilia, Italy). The primary active ingredients are terpenes and terpenoid molecules, with the largest constituent on a weight basis being thymol. The blend was tested in in vitro experiments at a concentration of 200 ppm of product. Stock solutions of BOT for in vitro experiments were prepared in 100% (v/v) ethanol and added to the basal culture medium ensuring that the final ethanol concentration was <1% (v/v). Adequate ethanol controls were included in the studies.

The human hepatocellular carcinoma cell line (**HepG2**) was acquired from ATCC (HB-8065; Virginia, USA). HepG2 cells were maintained in basal medium composed of Dulbecco’s modified Eagle’s medium supplemented with 10% fetal bovine serum, 1% l-glutamine, 1% non-essential amino acids, and 1% penicillin/streptomycin. Cells were incubated at 37 °C and 5% CO_2_ at 95% relative humidity.

#### Inflammatory challenge and IL-6 quantification.

 HepG2 cells were seeded in 4 different 24-well plates at a density of 8 × 10^4^ cells/well and allowed to grow until 90% confluence. Hepatocytes were then challenged for 8 d with basal medium containing LPS from *E. coli* O55:B5 (Merck KGaA) at 1 μg/mL, and the BOT-treated cells received the BOT at 200 ppm in the basal medium. The inflammatory challenge with LPS and BOT treatment was repeated on days 2 and 6 to ensure continuous stimulation over the 8-day study. Other than the treated and challenged group (**BOT+**), for each plate the experimental design included a negative control group maintained in basal medium (**CTR−**) and a positive control group maintained in basal medium and challenged with LPS (**CTR+**). Each treatment had 6 repetitions (*n* = 6).

On days 1, 3, 6, and 8, 1 plate was selected to collect supernatants for IL-6 quantification with Lumit IL-6 Human Immunoassay (Promega Corporation, Milan, Italy) measuring luminescence with Varioskan LUX (Thermo Fisher Scientific, Waltham, MA, USA).

#### Gene expression analysis.

On days 1 and 8, cells were washed once with DPBS and harvested for gene expression analysis. The 2 timepoints were selected to investigate an early and a late response to the LPS challenge. Gene expression was performed as described in our previous studies. Briefly, RNA was obtained with NucleoSpin RNA Kit (Macherey-Nagel, Düren, Germany) with DNase digestion according to the manufacturer’s instructions. Yield and purity of the extracted RNA were evaluated by measuring A230, A260, and A280 nm (µDrop Plate and Varioskan LUX, Thermo Fisher Scientific, Waltham, MA, USA). Then, reverse-transcription of the genetic material was performed by using iScript cDNA Synthesis Kit (Bio-Rad Laboratories, Hercules, CA, USA) according to the manufacturer’s instructions. Finally, cDNA was used as template for qPCR by using a CFX96 Real-Time PCR Detection System (Bio-Rad Laboratories) under the following conditions: 3 min at 95 °C, followed by 40 cycles of 95 °C for 10 s and 60 °C for 30 s. Reactions were prepared with iTaq Universal SYBR Green Supermix (Bio-Rad Laboratories), with primers for amplification reported in Table 1. The specificity of each reaction was evaluated by melting-curve analysis. Gene expression levels were normalized using 2 reference genes, ribosomal protein lateral stalk subunit P0 (**RPLP0**) and glyceraldehyde-3-phosphate dehydrogenase (**GAPDH**). Relative changes in gene expression were calculated with the 2−^ΔΔCt^ method (Livak and Schmittgen, 2001).

**Table 1.** Primers used in the current study for gene expression analysis

Function	Gene	Sequences (5´ →3´)	Product length (bp)	AN
Human primers (in vitro studies)				
Innate immune response	*TLR-4*	F: CCCTGAGGCATTTAGGCAGCTA R: AGGTAGAGAGGTGGCTTAGGCT	126	NM_003266.4
	*TNF-α*	F: TCTCGAACCCCGAGTGACAA R: TATCTCTCAGCTCCACGCCA	124	NM_000594.4
	*IL-1β*	F: AATCTGTACCTGTCCTGCGTGTT R: TGGGTAATTTTTGGGATCTACACTCT	78	NM_000576.3
	*IL-6*	F: AGCCCTGAGAAAGGAGACATGT R: AGGCAAGTCTCCTCATTGAATCC	141	NM_000600.2
	*IL-8*	F: GAGAGTGATTGAGAGTGGACCAC R: CACAACCCTCTGCACCCAGTTT	112	NM_000584.4
Oxidative stress response	*GPX-2*	F: CTCACTCTGCGCTTCACCAT R: TGCCCCGGAACGTATTGAAA	103	NM_002083.4
	*SOD*	F: GGAGATGTTACAGCCCAGATAG R: CGTTAGGGCTGAGGTTTGT	100	NM_001322819.2
	*CAT*	F: GTGCGGAGATTCAACACTGCCA R: CGGCAATGTTCTCACACAGACG	109	NM_001752.4
House keeping	*RPLP0*	F: GCAATGTTGCCAGTGTCTG R: GCCTTGACCTTTTCAGCAA	142	NM_001002.3
	*GAPDH*	F: TGCACCACCAACTGCTTAGC R: GGCATGGACTGTGGTCATGAG	87	NM_02046
Pig primers (in vivo study)				
Tight-junction integrity	*ZO-1*	F: CTCGTCGGGTGATCCTAAAA R: CGGTCTGCAGCATGTTTCTA	296	XM_003353439.2
	*CLD-1*	F: TGATGAGGTGCAGAAGATGC R: CCAGTGAAGAGAGCCTGACC	174	NM_001244539.1
	*OCCL*	F: TCGGACTATGCGGAGAGAGT R: TTTGAAGACGCCTCCAAGTT	200	NM_001163647.2
Innate immune response	*TLR-4*	F: GCCATCGCTGCTAACATCATC R: CTCATACTCAAAGATACACCATCGG	108	NM_001113039.2
	*TNF-α*	F: CCCTGGTACGAACCCATCTA R: TGAGGGGGTCTGAAGGAGTA	204	NM_214022.1
	*IL-6*	F: GAGAAAGGAGATGTGTGAGAAG R: GATTCTCATCAAGCAGGTCTC	145	NM_214399.1
	*IL-8*	F: TAGGACCAGAGCCAGGAAGA R: CAGTGGGGTCCACTCTCAAT	230	NM_213867.1
	*IFN-γ*	F: CCATTCAAAGGAGCATGGAT R: TGCAGGCAGGATGACAATTA	256	NM_213948.1
	*BD-2*	F: CCAGCTGGCTGCAGGTATTA R: ACTTGGCCTTGCCACTGTAA	149	NM_214442.2
	*BD-3*	F: CCTTCTCTTTGCCTTGCTCTT R: GCCACTCACAGAACAGCTACC	163	XM_021074698.1
Oxidative stress response	*GPX-2*	F: ACCCTCAGGTACGCTCACAC R: GCCTCGGAATGTGTTGAAAT	133	NM_001115136.1
	*SOD-1*	F: TCCATGTCCATCAGTTTGGA R: AGTCACATTGCCCAGGTCTC	131	NM_001190422.1
	*SOD-2*	F: TTTGGGGCTGTTTTTGTAGG R: TGATGGTTTGGGATGGTTTT	250	NM_214127.2
	*CAT*	F: CTGCCTGCAACGTTCTGTAA R: TTGGCATGCACAACTCTCTC	265	NM_214301.2
House keeping	*RPL4*	F: CAGCACTGAAAGCCAAATCA R: TTCTTCTGTGGTGGGCTTCT	200	XM_003121741.3

Abbreviations: AN, accession number; F, forward; R, reverse; TLR-4, toll-like receptor 4; TNFα, tumor necrosis factor α; IL-1β, interleukin 1β; IL-6, interleukin 6; IL-8, interleukin 8; IFN-γ, interferon γ; ZO-1, zonula occludens 1; CLD-1, claudin 1; OCCL, occludin; GPX-2, gluathione peroxidase 2; SOD, superoxide dismutase; CAT, catalase; BD-2, beta-defensin 2; BD-3, beta-defensin 3; RPLP0, ribosomal protein lateral stalk subunit P0; GAPDH, glyceraldehyde-3 phosphate dehydrogenase; RPL4, ribosomal protein L4.

#### ROS measurement.

To measure ROS production after an inflammatory challenge, in a separate experiment, HepG2 cells were seeded at a density of 1.0 × 10^4^ cells/well onto 96-well plates and maintained in basal medium. After reaching 90% of confluence, cells were treated with BOT or Vitamin C (VitC) for 24 h. Vitamin C 150 µM was used as a standard of antioxidant potential as already optimized in our previous study (Toschi et al., 2022). Then, challenge was performed with 1 μg/mL of *E. coli* O55:B5 LPS (Merck KGaA) for 24 h or 500 µM H_2_O_2_ for 1 h to stimulate ROS production. ROS were measured with CellROX Deep Red Reagent (Thermo Fisher Scientific, Milan, Italy) following manufacturer’s instructions. Fluorescence was measured with Varioskan LUX (Thermo Fisher Scientific, Waltham, MA, USA).

### In vivo *experiment*

#### Animals, experimental design, and diets.

Seventy-two newly weaned pigs (commercial crossbreds) with an average age of 24 d, balanced for barrows and gilts, and with an average body weight of 7.6 ± 0.4 kg, were selected from a commercial farm and moved to the University of Maryland’s animal facility. Pigs were allotted into a total of 24 pens (3’ × 6’) with 3 same-sex littermates. Pens were then assigned to 1 of the 3 treatments: non-challenged control fed a standard diet (CTR−, negative control, 8 pens), challenged control fed a standard diet (**CTR+**, positive control, 8 pens), and challenged treatment fed a standard diet supplemented with AviPower^®^5 (Vetagro S.p.A., Reggio Emilia), a proprietary blend of botanicals microencapsulated in a lipid matrix (**BOT+**, 1.5 g/kg of feed, 8 pens). Sex was balanced across treatments and pigs had ad libitum access to water and a mash nursery standard diet that met or exceeded NRC recommendations (NRC, 2012) and was manufactured by Form-A-Feed, Inc. (Steward, MN, USA). The standard diet contained porcine plasma and pharmacological zinc oxide to avoid confounding detrimental factors deriving from post-weaning diarrhea and eventual colibacillosis, but no antibiotics were added. The complete composition of the standard diet is shown in Table 2.

**Table 2.** Composition of basal diet

Item	Basal diet
Feedstuff, %	
Oatmeal	25.00
Whey	20.00
Soybean meal (46% crude protein)	17.00
Corn ground 6.8%	16.20
Deproteinized whey (permeate)	6.75
Fish meal	4.00
Porcine plasma	4.00
Vegetable oil	3.15
Vitamin–mineral premix (Pig Starter 50 Premix)	2.50
Salt	0.40
Calcium carbonate 38%	0.35
l-Lysine	0.34
dl-Methionine	0.21
l-Threonine	0.05
l-Valine	0.04
l-Tryptophan	0.02
Calculated composition	
ME, kcal/kg	3166
Crude protein, %	23.49
Crude fat, %	6.82
Crude fiber, %	2.00
SID Lys, %	1.70
Ca, %	0.68
STTD P, %	0.85
Zinc oxide, ppm	2390

Abbreviations: ME, metabolizable energy; SID, standardized ileal digestibility; STTD P, standardized total tract digestible phosphorus.

#### Experimental and sampling procedures.

Individual body weights were recorded initially and then weekly and average daily gain (**ADG**) and average daily feed intake (**ADFI**) per pen were calculated. Feed efficiency was then calculated as the ratio between ADFI and ADG per pen (**FCR**). On days 14 and 16, piglets in challenged groups were intraperitoneally injected with LPS from *E. coli* O55:B5 (Merck KGaA) at 30 µg/kg of body weight, or the same amount of sterile saline (0.9% NaCl) in the unchallenged control group. LPS type and doses were chosen basing on currently available scientific literature, aiming to exert a protracted mild challenge (Wyns et al., 2015). After each LPS injection, pigs were continuously monitored for 8 h, then checked every 8 h during the following days. Two hours after each LPS injection, all pigs experienced evident signs of lethargy, excessive salivation, and malaise with occasional vomiting and sporadic panting, all symptoms expected due to mild endotoxemia. All symptoms resolved approximately 6 h after each challenge in all pigs. However, 2 pigs in the CTR+ group died in response to the LPS challenge with signs of severe endotoxemia. Additionally, 1 pig in the BOT+ group had to be euthanized later in the study for issues not related to the experimental challenge protocol or dietary treatment.

On day 21 (1 animal per pen) and day 28 (all remaining animals), pigs were euthanized by penetrating captive bolt followed by exsanguination and tissues collected for subsequent analyses. Two jejunum segments (located 3 m from ileocecal valve) were collected from each pig and washed with saline: 1 was embedded and frozen in optimal cutting temperature medium (**OCT**, Fisher Scientific, New Hampshire, USA), while the second was dissected longitudinally, then gently scraped with a glass slide to collect the mucosa. Two samples of liver per pig were also collected. All tissues were immediately snap-frozen in liquid nitrogen and stored at −80 °C until further processing.

#### Gene expression analysis.

Jejunal mucosa and liver tissues were homogenized with FastPrep Advanced Bench-Top Lysis System (MP Biomedicals, CA, USA) using Lysing Matrix D in TRIzol Reagent (Thermo Fisher Scientific, MA, USA). Supernatant was obtained, mixed with chloroform, and then centrifuged. The colorless upper aqueous phase, containing solubilized RNA, was collected and stabilized in 70% ethanol. Total RNA extraction was continued using the RNeasy RNA extraction kit (QIAGEN, Hilden, Germany), following manufacturer’s instructions. Genomic DNA contamination was removed by lysis with DNase during the RNA extraction procedure. RNA yield and purity were verified by spectrophotometer measuring A260 and A280, with all samples showing purity values between 1.9 and 2.1. A total of 1 µg of RNA was reverse transcribed with iScript cDNA Synthesis Kit (Bio-Rad Laboratories) according to the manufacturer’s instructions, and obtained cDNA was quantified with Quant-iT Oligreen ssDNA Assay (Life Technologies, Oregon, USA). Real-time quantitative PCR was performed with iTaq Universal SYBR Green Supermix (Bio-Rad Laboratories) by using a CFX96 Real-Time PCR Detection System (Bio-Rad Laboratories) with the following thermocycling conditions: 30 min at 95 °C, followed by 40 cycles of 95 °C for 5 s and 60 °C for 30 s. To verify the absence of unspecific products, a melting-curve analysis was performed for all reactions. Gene expression was normalized using the housekeeping gene encoding porcine ribosomal protein L4 (**RPL4**). Relative changes in gene expression were calculated using the 2^−ΔΔCt^ method (Livak and Schmittgen, 2001). Porcine primers used for gene expression analysis were obtained from IDT (Iowa, USA), and their sequences are reported in Table 1.

#### ELISA protein analysis.

 Jejunal and liver samples from day 21 sacrificed animals were suspended in phosphate-buffered saline (**PBS**) and homogenized on ice using the tissue homogenizer Fisherbrand Bead Mill 24 Homogenizer (Fisher Scientific, Pennsylvania, USA). Samples were then centrifuged at 13,000 × *g* for 15 min, and supernatants collected and stored at −80 °C until further analysis.

The homogenized protein samples were used to measure total protein, TNFα, IL-6, IL-8, and IFN-γ concentrations. Total protein concentration was determined using the Pierce BCA Protein Assay Kit (Thermo Fisher Scientific, Massachusetts, USA) after appropriate sample dilution. Total protein concentrations were used to normalize concentrations of TNFα, IL-6, IL-8, and IFN-γ.

The concentration of cytokines was measured by using specific detection kits combined with DuoSet ELISA Ancillary Reagent Kit (R&D Systems, Minnesota, USA). TNFα was measured using the porcine TNFα Quantikine enzyme-linked immunosorbent assay (ELISA) Kit (R&D Systems), with a working range from 31.3 to 2,000 pg/mL. The concentration of IL-6 was measured using the IL-6 Quantikine ELISA Kit (R&D Systems), with a working range from 18.8 to 1,200 pg/mL. The concentration of IL-8 and IFN-γ were measured with IL-8 Quantikine ELISA Kit and IFN-γ Quantikine ELISA Kit, respectively (R&D Systems), with a working range from 62.5 to 4,000 pg/mL. After adequate sample dilution and after following manufacturer’s instructions, cytokines concentrations were determined.

#### Histologic analyses.

 Jejunal OCT blocks frozen during sampling were sectioned (5 µm thick) and mounted on glass slides. For histomorphology analyses, Hematoxylin & Eosin (**H&E**) staining was performed. Slides were fixed in 95% ethanol and 10% formalin, then stained with Harris Modified Hematoxylin (Fisher Chemical, New Hampshire, USA) for 30 s. After washing in water and 95% ethanol, slides were counterstained with Eosin Y (Fisher Chemical) for 25 s, then washed in 95% and 100% ethanol, and finally cleared in Clear-Rite 3 (Epredia Signature, Michigan, USA). For mast cell count and activation, Toluidine Blue (**TB**) staining was performed. Sections were fixed for 1 h in Carnoy’s fixative (60% ethanol, 30% chloroform, 10% glacial acetic acid). Then, slides were stained with 0.5% Toluidine Blue O (Fisher Chemical) in 0.5 N HCl in PBS for 45 min. After mounting, slides were digitalized with Axioscan 7 Microscope Slide Scanner (Carl Zeiss, Germany; USA). Measurements for crypt depth (**CD**), villi height (**VH**), and villi width (**VW**) were taken on H&E slides with Zeiss Zen 3.7 software (Carl Zeiss). Measurements were recorded at the same magnification level from 5 well-oriented villi for each slide (1 slide per animal). Mast cells analysis was performed on TB slides by using MIPAR 4.2.2 (Ohio, USA): mast cell count was automated by recognizing specific staining for mast cells, and degranulation was estimated by measuring cell eccentricity (e), assuming that activated mast cells tend to lose their circular shape (e ≈ 0) to display a more elongated profile (e ≈ 1). Mast cells with e ≥ 0.65 were considered degranulated and % of degranulation was calculated.

### Statistical analyses

For the in vitro experiments, the experimental unit was the well, with *n* = 6 for each group and data displayed as means ± SEM. All data were processed using GraphPad Prism v.10.0.0 (GraphPad Software, Inc., California, USA). IL-6 quantification, gene expression, and ROS measurement data were evaluated with One-Way ANOVA analysis with Tukey post-hoc test, comparing all experimental groups with each other within all timepoints of study and/or gene of interest. For the in vivo experiment, performance data were analyzed with SPSS 29.0.1.0 (IBM, New York, USA). The statistical unit was the pen, and values reported are EMMEANS ± SEM. Performance data were analyzed with ANCOVA, setting initial body weight as a covariate, with Bonferroni multiple comparisons post-hoc test. Gene expression, protein expression, and histologic data were reported as means ± SEM and analyzed with One-Way ANOVA with Tukey post-hoc test by using GraphPad Prism v.10.0.0 (GraphPad Software, Inc., California, USA), considering the pig as the experimental unit. For all the analyses, differences were considered significant when *P* ≤ 0.05, while tendencies were identified when 0.5 < *P* ≤ 0.1.

## Results

### In vitro *experiments*

#### IL-6 quantification.

**Figure 1.**
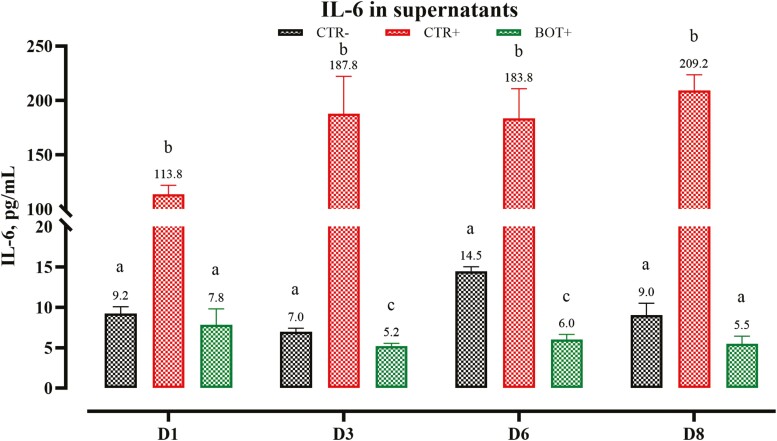
Quantification of IL-6 at different timepoints (days 1, 3, 6, and 8 after beginning of the challenge) in supernatants of HepG2 cells challenged for 8 d with *E. coli* O55:B5 LPS and treated with the blend of botanicals (BOT). CTR− = negative control; CTR+ = LPS challenge; BOT+ = LPS challenge with blend of botanicals. For each group, data are represented as means ± SEM of 6 different replicates. Within each timepoint, One-Way ANOVA analysis is performed with Tukey post-hoc test comparing all groups with each other. Different superscript letters indicate differences with *P* < 0.05.

Compared to negative control, CTR+ cells produced higher concentrations of IL-6 in the culture medium (*P* < 0.05), reaching a plateau on day 3 of challenge (Figure 1). The addition of BOT to the challenge cells prevented increased production of IL-6 (*P* < 0.05 vs. CTR+). BOT+ cells maintained IL-6 concentrations similar to or less than the CTR− (Figure 1).

#### Gene expression analysis.

**Figure 2.**
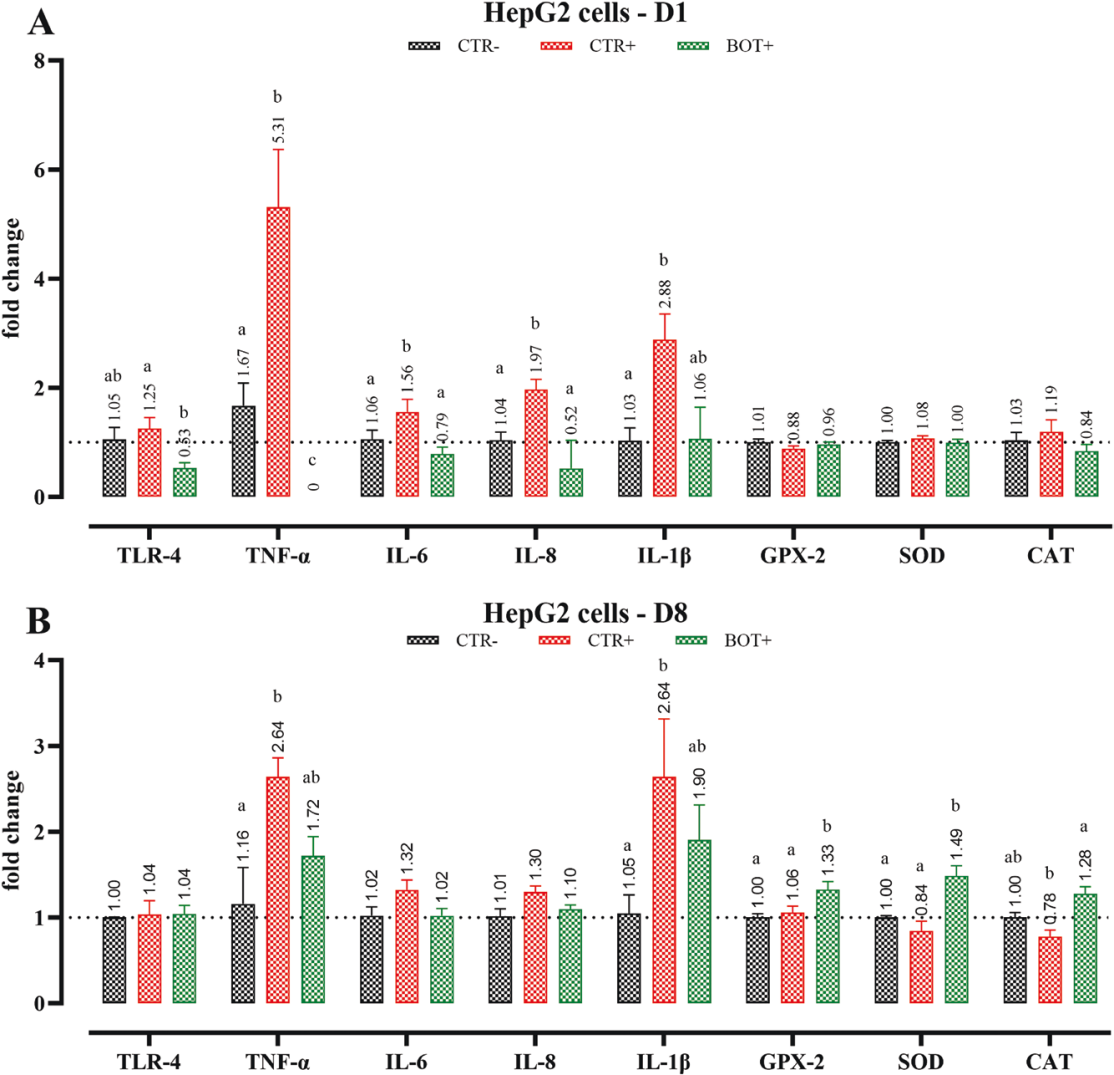
Gene expression analysis of HepG2 hepatocytes harvested on day 1 (D1, panel A) or day 8 (D8, panel B) treated with the blend of botanicals (BOT) and challenged for 8 d with *E. coli* O55:B5 LPS. CTR− = negative control; CTR+ = LPS challenge; BOT+ = LPS challenge with blend of botanicals. For each group, data are represented as means ± SEM. Within each marker assessed, One-Way ANOVA analysis is performed with Tukey post-hoc test comparing all groups. Different superscript letters indicate differences with *P* < 0.05.

On day 1, 24 h after the start of the challenge, the expression of TNF-α, IL-6, IL-8, and IL-1β was increased in the CTR+ group (*P* < 0.05), with the BOT treatment maintaining their mRNA levels at values equal to or lower than CTR− (Figure 2). Additionally, BOT decreased (*P* < 0.05) the expression of TLR-4 compared to CTR+. By the end of the study, on day 8, there were no longer differences in TLR-4 expression, but higher expression levels of TNF-α and IL-1β were detected in CTR+ group (*P* < 0.05), with BOT partially decreasing their expression. No variation in the mRNA concentrations of antioxidant enzymes was observed on day 1, but by the end of the experiment, GPX-2, SOD, and CAT showed higher expression in BOT+ group compared to CTR+ (*P* < 0.05).

#### ROS measurement.

**Figure 3.**
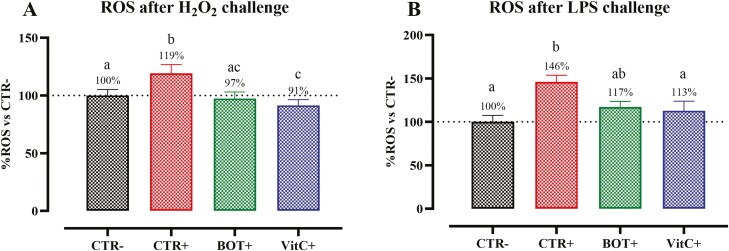
Reactive oxygen species (ROS) in HepG2 cells pretreated with the blend of botanicals (BOT) or Vitamin C (VitC) for 24h, and subsequently challenged for 1h with H_2_O_2_ (A) or for 24h with *E. coli* O55:B5 LPS (B). CTR− = negative control; CTR+ = H_2_O_2_/LPS challenge; BOT+ = H_2_O_2_/LPS challenge with blend of botanicals; VitC+ = H_2_O_2_/LPS challenge with Vitamin C. For each group, data are represented as means ± SEM of 8 different replicates. One-Way ANOVA analysis is performed with Tukey post-hoc test comparing all groups with each other. Different superscript letters indicate differences with *P* < 0.05.

The ROS levels of HepG2 cells treated 24 h with BOT and challenged for 1 h with H_2_O_2_ or 24 h with LPS are reported in Figure 3. Both challenges increased the levels of ROS, with LPS having a greater increase. Pretreatment with BOT allowed the control of ROS produced by H_2_O_2_ and LPS: BOT+ lowered ROS production compared to CTR+ (*P* < 0.05), keeping values similar to CTR− and VitC-treated cells (VitC+).

### In vivo *experiment*

#### Growth performance.

Table 3 displays the growth performance of pigs challenged with LPS and fed diets containing BOT. In the pre-challenge period (days 0 to 14), no significant differences were observed by BOT supplementation.

**Table 3.** Effect of LPS challenge on the growth performance of piglets challenged with *E. coli* O55:B5 LPS and supplemented with BOT treatment in the diet

Item	Treatment^1^				
	CTR−	CTR+	BOT+	SEM	*P*-value
BW, kg					
Day 0	7.7	7.8	7.4	0.4	0.676
Day 7	10.4	10.3	10.5	0.4	0.959
Day 14	14.4	13.6	13.9	0.5	0.535
Day 21	19.9^a^	16.6^b^	17.3^ab^	0.6	0.010
Day 28	25.7	20.9	23.3	1.1	0.055
ADG, g/d/pig					
Days 0 to 7	374	391	432	32	0.362
Days 7 to 14	578	592	570	46	0.929
Days 14 to 21	686^a^	488^b^	571^ab^	45	0.023
Days 21 to 28	699	679	768	33	0.071
Days 0 to 14	507	457	488	31	0.530
Days 14 to 28	726	608	696	37	0.114
Days 0 to 28	617	521	593	31	0.091
ADFI, g/d/pig					
Days 0 to 7	424	376	415	15	0.064
Days 7 to 14	696	710	695	34	0.934
Days 14 to 21	736	776	758	65	0.923
Days 21 to 28	874	928	1,058	79	0.205
Days 0 to 14	550	530	545	19	0.729
Days 14 to 28	800	837	881	61	0.662
Days 0 to 28	656	657	670	42	0.957
FCR					
Days 0 to 7	1.14	1.03	1.01	0.10	0.361
Days 7 to 14	1.20	1.20	1.22	0.10	0.819
Days 14 to 21	1.11^a^	1.61^b^	1.33^ab^	0.12	0.038
Days 21 to 28	1.20	1.40	1.34	0.05	0.211
Days 0 to 14	1.08	1.16	1.13	0.04	0.300
Days 14 to 28	1.14^a^	1.40^b^	1.29^ab^	0.05	0.031
Days 0 to 28	1.06	1.26	1.13	0.04	0.178

^1^Treatments: CTR− = negative control, without challenge, fed standard diet; CTR+ = positive control, with LPS challenge on days 14 and 16, fed standard diet; BOT+ = pigs challenged with LPS on days 14 and 16, fed with diet containing the blend of botanicals (1.5 g/kg).

^a,b^Values within a row with different superscripts differ significantly at *P *< 0.05.

Abbreviations: SEM, standard error of the mean; BW, body weight; ADG, average daily gain; ADFI, average daily feed intake; FCR, feed conversion ratio.

The LPS challenge significantly reduced the ADG of pigs compared to CTR− (−198 g) in the week after the challenge (days 14 to 21), resulting in a lower BW (−3.3 kg; *P* < 0.05) and a higher FCR (+0.50 points; *P* < 0.05). During the same period, BOT+ piglets had BW, ADG, and FCR intermediate to those of CTR− and CTR+ piglets. During the last week of the study (days 21 to 28), the CTR+ group still had a numerically lower ADG compared to the CTR−, while BOT+ tended to improve ADG compared to the other experimental groups (*P* < 0.1), leading to an average final body weight intermediate to the CTR− and CTR+ groups. Considering the entire post-challenge period (days 14-28), the LPS challenge numerically reduced ADG (-118 g) and significantly increased FCR (+0.26 points) compared to CTR−, while the supplementation of BOT ameliorated this reduced performance. Over the entire post-challenge period, CTR+ pigs had lower ADG compared to the other experimental groups (*P* < 0.1).

#### Gene expression analysis.

**Figure 4.**
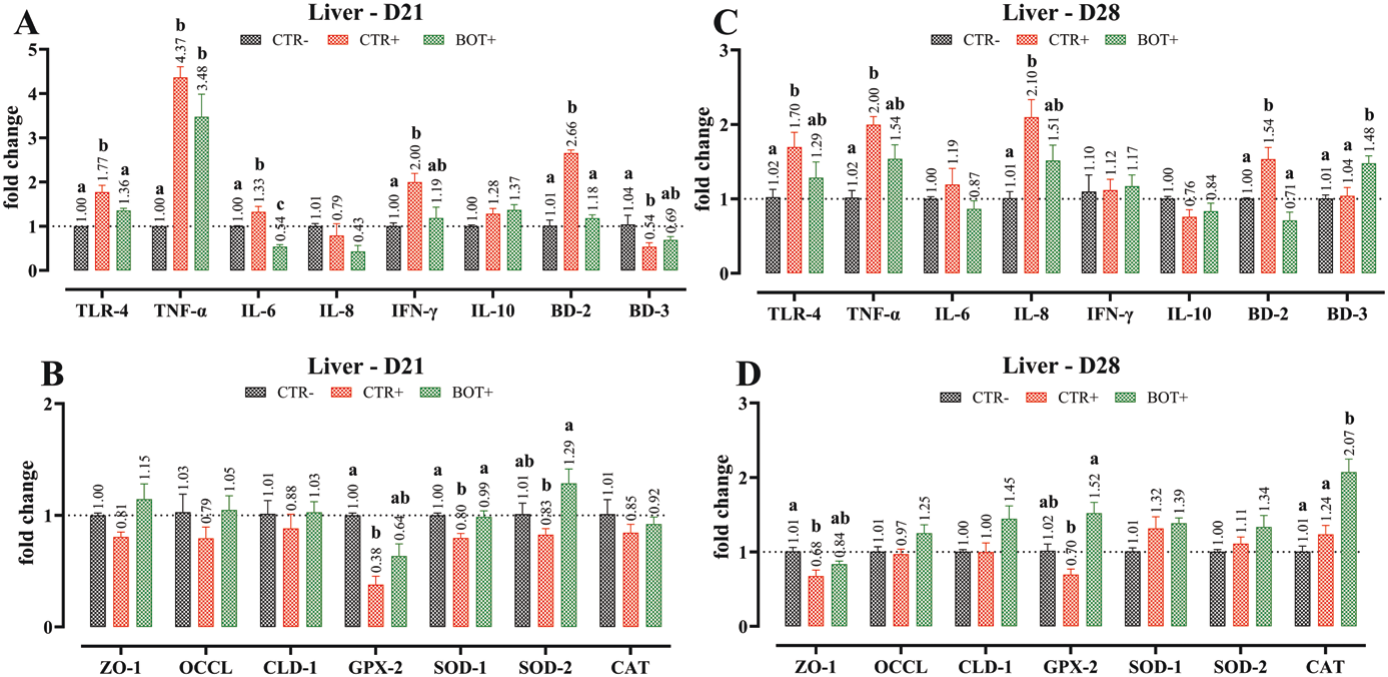
Gene expression analysis of liver samples collected at day 21 (D21, panels A and B) or day 28 (D28, panels C and D) from weaning piglets supplemented with the microencapsulated blend of botanicals (BOT) in the feed and challenged with *E. coli* O55:B5 LPS on days 14 and 16. CTR− = negative control; CTR+ = LPS challenge; BOT+ = LPS challenge with blend of botanicals. For each group, data are represented as means ± SEM. Within each marker assessed, One-Way ANOVA analysis is performed with Tukey post-hoc test comparing all groups. Different superscript letters indicate differences with *P* < 0.05.

LPS stimulation increased the expression of several inflammatory parameters in liver samples. In particular, LPS challenge significantly increased the expression of TLR-4 and TNF-α at days 21 and 28, with the expression of IL-6 and INF-γ particularly at day 21, and the expression of IL-8 at day 28 (*P* < 0.05, Figure 4). The addition of BOT to the diet of challenged pigs lowered the expression of TLR-4, TNF-α, IL-6, and INF-γ on day 21, and a reduced expression of TNF-α and IL-8 on day 28 (*P* < 0.05). The CTR+ animals had higher expression of BD-2 in the liver (*P* < 0.05), and the supplementation of BOT reduced its levels closer to CTR−. On the contrary, BD-3 was reduced by the LPS challenge of the CTR+ pigs on day 21, but it was restored at normal levels on day 28, and BOT supplementation significantly improved its expression at both timepoints (*P* < 0.05). On day 21, the mRNA levels of genes related to liver integrity (ZO-1, OCCL, and CLD-1) were not affected. However, on day 28, BOT+ animals had higher expression of ZO-1 compared to the CTR+ group (*P* < 0.05). The LPS challenge also decreased the hepatic transcription of genes related to the oxidative stress response, with significantly lower levels of GPX-2 (days 21 and 28), SOD-1, and SOD-2 (day 21 only). Supplementation of BOT improved the expression of all these antioxidant enzymes (*P* < 0.05). Finally, on day 28, the expression of CAT was significantly increased by BOT.

**Figure 5.**
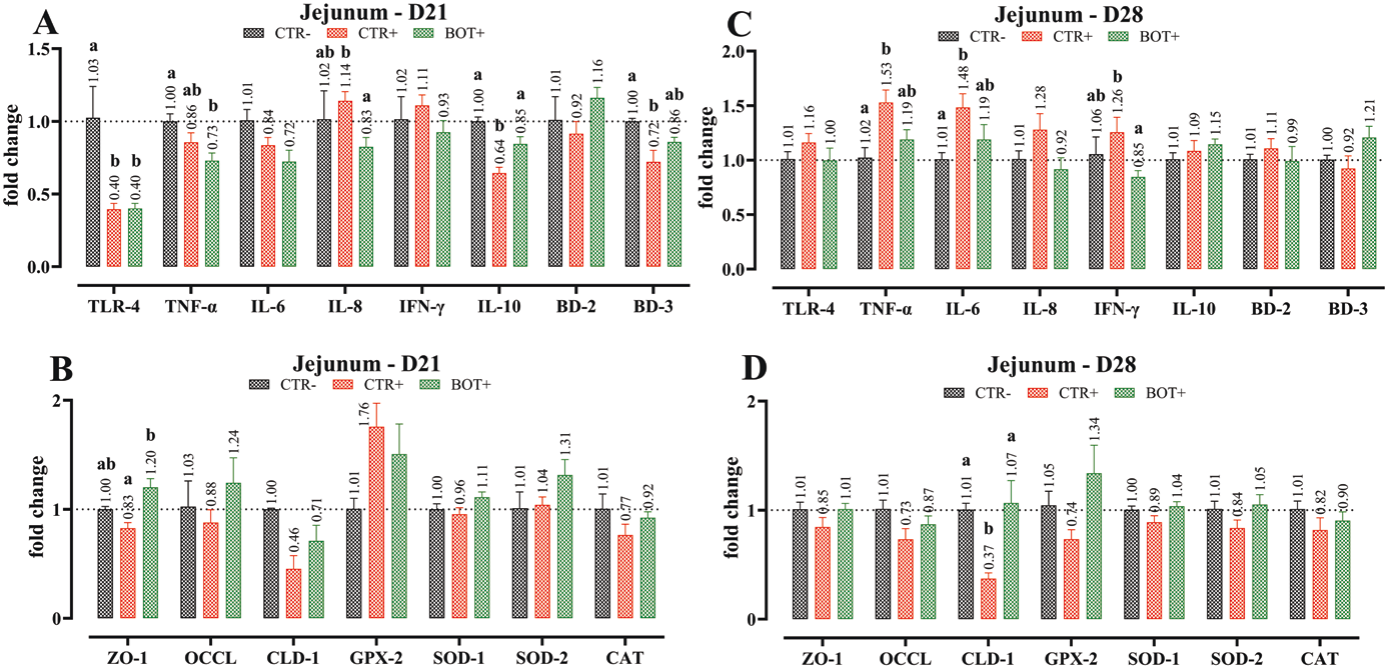
Gene expression analysis of jejunum samples collected at day 21 (D21, panels A and B) or day 28 (D28, panels C and D) from weaning piglets supplemented with the microencapsulated blend of botanicals (BOT) in the feed and challenged with *E. coli* O55:B5 LPS on days 14 and 16. CTR− = negative control; CTR+ = LPS challenge; BOT+ = LPS challenge with blend of botanicals. For each group, data are represented as means ± SEM. Within each marker assessed, One-Way ANOVA analysis is performed with Tukey post-hoc test comparing all groups. Different superscript letters indicate differences with *P* < 0.05.

Figure 5 reports the results of the gene expression analysis of jejunal samples. After the initial downregulation at day 21 (*P* < 0.05), no differences were seen for TLR-4 expression in jejunum samples. The LPS challenge increased the expression of pro-inflammatory cytokines. On day 28, TNF-α, IL-6, and IFN-γ were increased with LPS challenge (*P* < 0.05), and BOT treatment had mRNA levels closer to CTR−. On day 21, BD-3 showed a lower expression in the CTR+ group (*P* < 0.05), which was partially restored by BOT. In examining some of the intestinal tight-junction proteins, on day 21 the expression of ZO-1 was increased with BOT, while the LPS challenge reduced its levels (*P* < 0.05). A similar pattern was also seen for CLD-1 on day 28. No significant differences were found in gene expression data related to ROS detoxification in the jejunum.

#### ELISA protein analysis.

**Figure 6.**
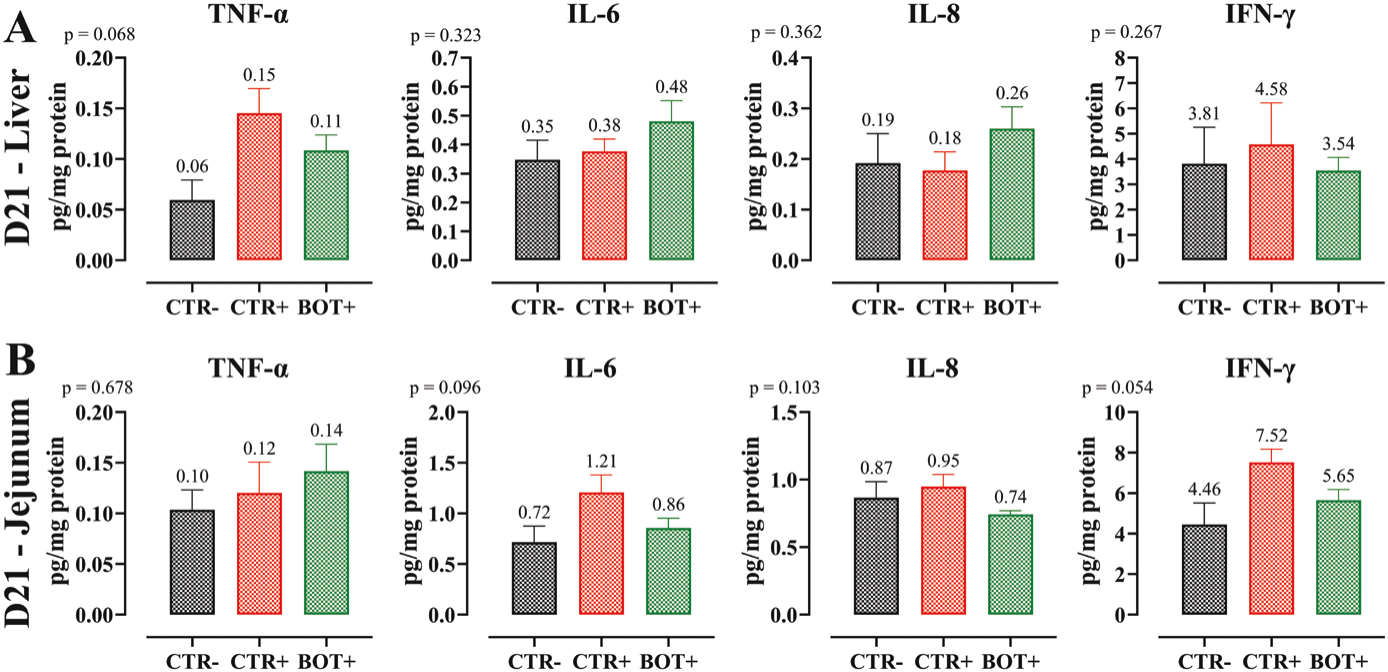
ELISA protein analysis of liver (panel A) and jejunum (panel B) samples collected at day 21 (D21) of the study from weaning piglets supplemented with the microencapsulated blend of botanicals (BOT) in the feed and challenged with *E. coli* O55:B5 LPS on days 14 and 16. CTR− = negative control; CTR+ = LPS challenge; BOT+ = LPS challenge with blend of botanicals. For each group, data are represented as means ± SEM. For each marker, One-Way ANOVA analysis is performed with Tukey post-hoc test comparing all groups. The *P*-value of each analysis is reported on the upper-left corner of each graph.

In general, no statistically significant differences were observed in pro-inflammatory cytokines, but a tendency (*P* < 0.1) was observed for TNF-α protein amount in liver, where BOT+ had an intermediate concentration between CTR+ and CTR− (Figure 6). In the jejunal samples, IL-6 and IFN-γ tended to display lower protein abundance in BOT+ and CTR− groups compared to CTR+ (*P* < 0.1).

#### Histologic analyses.

 In response to LPS challenge, pigs tended to have a higher jejunal CD (*P* < 0.1) and a significantly lower VH:CD ratio on day 21 (*P* < 0.05), as shown in Table 4. The supplementation of the BOT treatment restored these parameters to levels similar to the negative control. A similar effect was also seen among the histologic analysis of the jejunal samples collected at day 28, which displayed a reduction in CD and increase of VH:CD when animals were fed with BOT in the diet compared to CTR+ (*P* < 0.05).

**Table 4.** Effect of LPS challenge on intestinal morphology parameters of piglets challenged with *E. coli* O55:B5 LPS and supplemented with BOT treatment in the diet

Item	Treatment^1^				
	CTR−	CTR+	BOT+	SEM	*P*-value
Day 21					
Villi Height (VH, µm)	505	452	519	33	0.312
Villi Width (VW, µm)	124	133	127	8	0.827
Crypt Depth (CD, µm)	265	323	270	15	0.056
VH:CD ratio	1.93^a^	1.40^b^	1.95^a^	0.05	0.001
Day 28					
Villi Height (VH, µm)	519	471	528	19	0.106
Villi Width (VW, µm)	131	131	136	5	0.685
Crypt Depth (CD, µm)	271^a^	308^b^	255^a^	9	0.001
VH:CD ratio	1.92^a^	1.56^b^	2.08^a^	0.05	0.001

^1^Treatments: CTR− = negative control, without challenge, fed standard diet; CTR+ = positive control, with LPS challenge on days 14 and 16, fed standard diet; BOT+ = pigs challenged with LPS on days 14 and 16, fed with diet containing the blend of botanicals (1.5 g/kg).

^a,b^Values within a row with different superscripts differ significantly at *P *< 0.05.

Abbreviation: SEM, standard error of the mean.

TB staining was performed to count mast cells and visualize their degranulation (Table 5). Although no differences were detected at both timepoints in the number of mast cells in the jejunum, on day 21 the percentage of activated and degranulated mast cells, despite not being different between the 2 control groups, was significantly reduced in BOT+ compared to CTR+ piglets, that only received the LPS challenge (*P* < 0.05).

**Table 5.** Effect of LPS challenge on mucosal mast cell numbers and activation in piglets challenged with *E. coli* O55:B5 LPS and supplemented with BOT treatment in the diet

Item	Treatment^1^				
	CTR−	CTR+	BOT+	SEM	*P*-value
Day 21					
Mast cells count, cells/µm^2^	30	32	19	11	0.664
Activated mast cells, %	38^ab^	47^b^	33^a^	2	0.025
Day 28					
Mast cells count, cells/µm^2^	43	29	39	10	0.417
Activated mast cells, %	40	43	39	2	0.370

^1^Treatments: CTR− = negative control, without challenge, fed standard diet; CTR+ = positive control, with LPS challenge on days 14 and 16, fed standard diet; BOT+ = pigs challenged with LPS on days 14 and 16, fed with diet containing the blend of botanicals (1.5 g/kg).

^a,b^Values within a row with different superscripts differ significantly at *P *< 0.05.

Abbreviation: SEM, standard error of the mean.

## Discussion

Weaning stress causes bacterial LPS translocation from the intestine to the liver in piglets. The combination of both physical and psychological stressors that occur at weaning, generate substantial damages to the intestine, and may trigger a persistent inflammatory and oxidative status that impairs epithelial integrity and may allow the colonization of undesired pathogens like Enterotoxigenic *E. coli*, which may further damage the intestinal epithelium with their toxins (Syed and Dubreuil, 2012; Mukiza and Dubreuil, 2013). The result of these issues is a reduced gut barrier function (Kim and Duarte, 2021), which allows the translocation of immunogenic bacterial components such as LPS (Dubreuil, 2017; Anand and Mande, 2022). Once translocated into the bloodstream, LPS is rapidly bound by LBP. The complex travels through the mesenteric veins to the liver, where the liver attempts to convert LPS into non-reactive forms. It does so by deploying Kupfer cells to deacetylate, and hepatocytes to dephosphorylate the LPS (Guerville and Boudry, 2016). During this process, LPS activates the liver’s innate immune response, causing the secretion of cytokines and the production of ROS (Su, 2002). With both liver cells and extrahepatic tissues, the LPS/LBP complex is recognized by TLR-4, the main LPS receptor of animal cells, which activates a signal transduction cascade that involves MyD88 and TAK kinase activation. These kinases phosphorylate and eliminate IkB, allowing NF-kB translocation into cell nucleus, where it triggers the transcription of inflammation- and oxidation-related genes (Medzhitov, 2001; Raetz and Whitfield, 2002).

Our in vitro results on hepatocytes confirm this mechanism of action. We successfully developed a prolonged LPS challenge model on HepG2 cell line in vitro, which immediately responded to the immune stimulation by activating the expression of cytokines, and maintaining it overtime after recurring LPS administration, as confirmed by PCR results. In the case of IL-6, despite a 0.5-fold increase in the mRNA levels of the cytokine, we were able to measure an increased secretion of the protein, which was maintained at significantly higher concentrations over the course of the study, compared to the other experimental groups. This confirms that, in the case of IL-6, even a moderate change in the gene expression may translate into a biologically meaningful increase in protein secretion. The H_2_O_2_ and LPS challenges increased ROS levels in hepatic cells, demonstrating how inflammation and oxidation are closely related, and part of the wider stress response in liver hepatocytes. In weaning pigs, excessive and/or prolonged liver stress from inflammatory activation and ROS production can generate long-term detrimental consequences, such as the establishment of subclinical chronic inflammation, the appearance of liver damage, and the onset of metabolic disorders that markedly affect animal growth. This all results in making the pigs more susceptible to a variety of illnesses (Cray et al., 2009; Pastorelli et al., 2012; Liehr et al., 2017).

Botanicals are nature-derived molecules that have been shown to exert relevant effects on inflammation, oxidative stress, and microbiota composition (Rossi et al., 2020). They are now being widely considered in pig production for their potential to replace or complement the action of no-longer sustainable tools, such as antibiotics and pharmacological doses of zinc oxide (Bonetti et al., 2021, 2023a). One strategy to increase the efficacy of botanicals is to combine those with diverse mechanisms of action to obtain a mixture with a synergistic activity (Toschi et al., 2020a). We have previously demonstrated that a blend of selected botanicals was effective in modulating acute and chronic enterocyte stress generated by LPS and ETEC in vitro through the control of host-pathogen interaction and the inflammatory activation of enterocytes (Bonetti et al., 2023b).

Because the liver is tightly connected to the gastrointestinal tract and is one of the main metabolic organs that responds to stimuli coming from the intestine (Szabo et al., 2010), we wanted to assess—in similar in vitro conditions—if BOT was able to ameliorate inflammatory and oxidative stress in hepatocytes. Our experiments confirmed the ability of BOT to significantly reduce the secretion of the pro-inflammatory cytokine IL-6 during an LPS challenge. This was accompanied by the modulation of the expression of several other pro-inflammatory cytokines, which are all part of the first-line response to LPS (Tanaka et al., 2014; Robinson et al., 2016). Overall, our data showed that the BOT mode of action is likely connected to the modulation of markers that are all controlled by TLR-4 activation and NF-kB translocation into the nucleus of cells (Kuzmich et al., 2017). This is likely a consequence of BOT components ability to interact with several mediators of the inflammatory cascade. Several botanicals, like thymol, are particularly effective in inhibiting NF-kB phosphorylation, probably due to a direct interaction with NF-kB (Liang et al., 2014; Nagoor Meeran et al., 2017; Laurindo et al., 2023) and a disruption of the functionality of kinases responsible for the initial activation of NF-kB (Liang et al., 2014). BOT components also possess strong antioxidant action, as demonstrated by ROS measurements in H_2_O_2_- and LPS- challenged HepG2 cells. This is not only due the direct antioxidant activity that many botanicals naturally possess (Hashemi and Davoodi, 2011; Manuelian et al., 2021), but also to their capacity to trigger Nrf2, a transcription factor that promotes the synthesis of several antioxidant enzymes (Jung and Kwak, 2010). For example, by accelerating Keap1 degradation (a Nrf2 inhibitor), thymol can stimulate Nrf2 translocation into the cell nucleus and the transcription of antioxidant responsive elements (Stefanson and Bakovic, 2014; Zou et al., 2016). Our data demonstrated that the expression of GPX, SOD, and CAT was significantly increased after 8 d of BOT treatment in HepG2 cells, while the LPS challenge was ongoing. It is possible that the higher expression of these enzymes could indicate a higher scavenging activity against ROS produced by an inflammatory activation. Future studies could explore this aspect to better elucidate the BOT mode of action. Nevertheless, by controlling oxidative stress at different stages, BOT components likely limit the ROS-dependent NF-kB activation. This further modulates the inflammatory response and the resulting ROS production it triggers, disrupting a harmful pro-inflammatory loop (Lugrin et al., 2014; Wardyn et al., 2015).

Considering the positive effects of BOT in different intestinal and liver in vitro challenge models, our research continued by investigating and confirming its beneficial actions in an in vivo challenge model. This model was designed to mimic chronic inflammation in weaning piglets. Our model consisted of two, 48-h-apart, intraperitoneal (IP) injections of 30 µg/Kg BW *E. coli* O55:B5 LPS. Our goal was to trigger a mild inflammatory state that would closely resemble what occurs in practical production settings (Martínez-Miró et al., 2016). The LPS injections effectively impaired the growth performance parameters of weaning piglets. The CTR+ animals had significantly lower BW and ADG 7 days after the start of the challenge compared to the negative control, with trends still present at 28 days. Moreover, there was a significant decrease in feed conversion efficiency, even though the challenge itself did not affect the feed intake. The lack of effect of the LPS challenge on feeding behavior may be attributed to the temporal progression of the challenge itself. While the observed symptoms were consistent with a status of mild endotoxemia (Wyns et al., 2015), lethargy and malaise were transient, lasting only a few hours. This ultimately allowed pigs to quickly resume their regular feed intake, as previously demonstrated in similar studies (Wright et al., 2000). It is likely that LPS-challenged pigs redirected nutrients away from growth to support the LPS-prompted immune response. Previous studies support this by showing reduced metabolizable energy available for growth and less fat and protein deposition in similar settings (Campos et al., 2014; Huntley et al., 2018). It could be hypothesized that this shift was reduced by the addition of BOT in the diet of challenged pigs. The bioactive compounds inside BOT significantly improved BW, ADG, and FCR in the week after the challenge, with values closer to the negative control, and helped animals to recover, resulting in an overall tendency to maintain growth performance parameters despite the LPS stress.

The IP challenge utilized in this study allows LPS to be drained by mesenteric capillaries to the vena cava, using the same pathway of the leaky-gut-derived LPS to reach the liver (Konturek et al., 2018; Ringseis and Eder, 2022). In the liver, LPS is partially detoxified, but also elicits an immune response, with long-lasting effects. Our gene expression data from liver samples showed a significant upregulation of TLR-4, TNF-α, IL-6, IL-8, and IFN-γ up to 14 days after the challenge, displaying a prolonged immune activation at the mRNA level to discrete LPS injections. This in vivo data was in strong agreement with the data from our in vitro experiments on HepG2 cells. Even if only TNFα protein concentration tended to be increased in CTR+ pigs compared to CTR−, the variations in genetic expression of cytokines still suggest a modulation of the liver inflammatory tone. The difference in the magnitude of responses to LPS between hepatocytes in vitro and liver tissues in vivo, especially for IL-6 protein expression, could be related to the intrinsic dissimilarities between the two challenge models. In particular, two factors may have affected this variation: the differences in the timing of analyses (closer to challenge in vitro, farther from challenge in vivo), and the direct exposure of hepatocytes to LPS in vitro compared to the varied cellular populations (like Kupffer cells) that participate in the hepatic detoxification of LPS in vivo (Guerville and Boudry, 2016). This latter aspect might have partially blunted the inflammatory response in the liver compared to cultured hepatocytes. Nevertheless, as confirmed by our study, the LPS challenge and protracted liver inflammation modulated jejunal mucosa cytokine expression. Overall, this response suggests a certain degree of intestinal homeostasis perturbation, that resulted in an impairment of ZO-1, OCCL, and CLD-1 expression, confirming the close relationship between inflammation and intestinal permeability (Szabó et al., 2023). The disruption of the gut structure was also evident from the impairment of VH:CD ratio, mainly driven by an increased depth of intestinal crypts. The intestinal crypts usually undergo this morphology shift when they need to support the re-establishment of the intestinal barrier after stress (Liu, 2015; Zheng et al., 2021).

The supplementation of BOT in the diet of weaning piglets was able to counteract the negative impact of LPS-derived inflammation. At the intestinal level, BOT improved intestinal morphology due to the modulation of the innate immune response. This was shown by the reduction of the gene expression and the modulation of protein concentrations of certain pro-inflammatory cytokines, and the decreased mast cell activation compared to CTR+ pigs. In this context, it could be speculated that, by limiting the LPS-derived disruption of the intestinal barrier integrity, BOT reduced the translocation of undesirable antigens and compounds, thus lowering the “inflammatory load”—coming from the intestine—that the liver would need to detoxify. Besides acting in the intestine, it is well recognized that a non-negligible fraction of the BOT can be absorbed, reaching the liver as an intermediate step before being excreted (Zeng et al., 2015; Horky et al., 2019). Even if they can undergo metabolization processes, the components of BOT, and their metabolites, have beneficial properties, as demonstrated by our liver gene expression results. Our data showed decreased mRNA levels of several pro-inflammatory markers, alongside an increase in antioxidant enzyme expression. Taken together, the in vivo findings can support the BOT dualistic mechanism of action already proposed in vitro, involving the interference and downregulation of NF-kB pathway, while triggering Nrf2 activation. An additional confirmation of this effect comes from the in vivo expression data of beta-defensins: BOT did not increase BD-2 expression, a defensin whose stimulation is NF-kB- and inflammation-dependent (Vora et al., 2004), while it still enhanced BD-3 levels, whose synthesis can be enhanced by botanicals through NF-kB-independent pathways (Sechet et al., 2018).

The molecular interplay between the bioactive ingredients in BOT and the innate immune cellular mediators that compose the gut-liver axis further explains the improvements in the growth performance of treated piglets. The BOT modulated inflammation and reduced oxidation, allowing the utilization of a higher amount of nutrients for productive growth, rather than supporting immune reactions (Campos et al., 2014).

## Conclusion

In conclusion, this study demonstrated that BOT was effective in controlling stress at the liver level, both in vitro and in vivo. The BOT displayed a multifaceted mechanism of action, related to the modulation of inflammatory activation and the control of oxidative stress, thus unveiling a wide range of possible targets to combat stress-related decreases in animal performance. In our whole animal model, BOT was also able to act at the intestinal level by ensuring the maintenance of a better intestinal morphology and barrier integrity. These effects on overall animal health likely contributed to reduced immune activation and the energy expenditure needed to sustain it. This allowed the pigs to maintain their performance. Our data supports the utilization of BOT in piglets at weaning to prevent the damage derived from an excessive inflammatory response and to maintain improved overall health. Future studies should further investigate the mode of action of BOT by confirming its molecular activity and exploring the relationship between the measured biological effects and their in vivo physiologic relevance. Additionally, other in vivo trials in pigs should be addressed towards the investigation of BOT in other challenge models that involve the infection by pathogens or where animals are naturally exposed to multiple harmful stimuli.

## Abbreviations

ADFI: average daily feed intake
ADG: average daily gain
BD: beta-defensin
BOT: blend of botanicals
BW: body weight
CAT: catalase
CLD: claudin
ETEC: enterotoxigenic *Escherichia coli*
FCR: feed conversion ratio
GPX: glutathione peroxidase
IFN: interferon
IkB: inhibitory kappa beta
IL: interleukin
IP: intraperitoneal
Keap1: kelch-like ECH-associated protein 1
LBP: lipopolysaccharide binding protein
LPS: lipopolysaccharide
ME: metabolizable energy
MyD88: myeloid differentiation primary response 88
NF-kB: nuclear factor kappa beta
OCCL: occludin
ROS: reactive oxygen species
SOD: superoxide dismutase
SID: standardized ileal digestibility
STTD P: standardized total tract digestible phosphorus
TAK: transforming growth factor beta-activated kinase
TLR: toll-like receptor
TNF: tumor necrosis factor
ZO: zonula occludens
